# Long-Acting Injectable Drugs for HIV-1 Pre-Exposure Prophylaxis: Considerations for Africa

**DOI:** 10.3390/tropicalmed7080154

**Published:** 2022-07-29

**Authors:** Enos Moyo, Grant Murewanhema, Godfrey Musuka, Tafadzwa Dzinamarira

**Affiliations:** 1 Medical Centre Oshakati, Windhoek P.O. Box 3785, Namibia; moyoenos@gmail.com; 2 Faculty of Medicine and Health Sciences, University of Zimbabwe, Harare P.O. Box MP167, Zimbabwe; gmurewanhema@yahoo.com; 3 International Initiative for Impact Evaluation, Harare P.O. Box 7118, Zimbabwe; gmusuka@3ieimpact.org; 4 ICAP at Columbia University, Kigali P.O. Box 28, Rwanda; 5 School of Health Systems and Public Health, University of Pretoria, Pretoria 0002, South Africa

**Keywords:** long-acting cabotegravir injection, preexposure prophylaxis, HIV-1, Africa

## Abstract

Sub-Saharan Africa carries the highest burden of HIV-1 and AIDS. About 39% of all new infections in the world in 2020 were in this region. Oral PrEP was found to be very effective in reducing the risk of HIV-1 transmission. However, its effectiveness is highly dependent on users adhering to the drugs. The availability of long-acting injectable PrEP that eliminates the need for a daily pill may increase PrEP uptake and adherence in people who struggle to adhere to oral PrEP. The USA’s FDA approved long-acting cabotegravir (CAB-LA) for PrEP of HIV-1 in December 2021. In this review, we discussed the implementation challenges to the successful roll-out of CAB-LA in Africa and measures to address these implementation challenges. Some health system-level challenges include the cost of the drug, its refrigeration requirement, and the shortage of healthcare providers trained to administer parenteral medicines. In contrast, client challenges include lack of knowledge, accessibility of the drug, side effects, stigma, and lack of family and community support. These challenges can be addressed by several measures emanating from lessons learned from the successful implementation of ART, oral PrEP, and immunization in the continent. Some steps include advocating for waiving of CAB-LA patent licence, conducting demonstration projects in Africa, promoting the use of renewable energy sources such as solar energy, healthcare provider training, task shifting, community engagement, client education, and implementing adherence promotion strategies.

## 1. Introduction

Since the first cases of HIV-1 were diagnosed in 1981, the World Health Organization (WHO) has estimated that 79.3 million [55.9–110 million] people have been infected with the HIV-1 virus and 36.3 million [27.2–47.8 million] people have died of HIV-1 at the end of 2020. About 38 million people were living with HIV-1 (PLHIV) globally in 2020. Of the PLHIV globally, 84% knew their status, and about 28 million were accessing antiretroviral therapy (ART) in 2021. In the same year, 1.5 million people became newly infected with HIV. Sub-Saharan Africa carries the highest burden of HIV-1 and AIDS. Of all the PLHIV globally, 20.6 million live in sub-Saharan Africa. Of the PLHIV in this region, 16 million were accessing ART in 2020, which is 77% of PLHIV. About 670,000 people became newly infected with HIV-1 in sub-Saharan Africa in 2020, which translates to 39% of all new infections worldwide. In sub-Saharan Africa, women and girls accounted for 63% of all new infections [1]. PLHIV who do not know their status, PLHIV who know their status but are not yet on ART, and PLHIV who are on ART but are not virally suppressed, are likely to be driving the epidemic [2]. A study conducted in eight African countries revealed that all countries had more than 85% viral suppression except for Cote d’Ivoire only [3]. To reduce the transmission of HIV-1 in sub-Saharan Africa, combination strategies should be implemented. Combination strategies include behavioral, biomedical, and structural strategies [2].

Biomedical interventions include voluntary medical male circumcision (VMMC), antiretroviral treatment as prevention (TasP), prevention of mother-to-child transmission (PMTCT), preexposure prophylaxis (PrEP), and post-exposure prophylaxis (PEP) [4]. WHO recommended the use of PrEP for HIV-1 prevention in 2015. This recommendation followed the results of the Preexposure Prophylaxis Initiative (iPrEx) trial [5], which showed a 44% reduction in HIV-1 infection in those taking tenofovir/emtricitabine (TDF/FTC). Additional evidence from the Preexposure prophylaxis to prevent the acquisition of HIV-1 infection (PROUD) and the On-Demand Antiretroviral Pre-exposure Prophylaxis for HIV Infection in Men Who Have Sex With Men (IPERGAY) studies reported an 86% reduction in the risk of HIV-1 transmission in people receiving TDF/FTC. The iPrEx study showed that HIV-1 transmission could be reduced by up to 99% with full adherence. WHO recommended that PrEP be considered for any individual at risk of getting infected with HIV-1 [5]. The WHO updated the PrEP guidelines in 2019 to include the option of on-demand PrEP for men who have sex with men (MSM).

On-demand PrEP consists of a double dose of TDF/FTC 2–24 h before sex, followed by one dose at 24 h and another at 48 h after the first. In the on-demand option, if sexual activity continues, a single dose can be taken daily until two days after the last sexual act. In 2021, WHO recommended the dapivirine vaginal ring as an additional HIV-1 prevention choice for cisgender women. In 2016, the United Nations General Assembly set a global target of 3 million oral PrEP users by 2020. In the African region, 81% of the countries had adopted WHO PrEP recommendations by 2019. However, not many countries that adopted the recommendations are implementing PrEP services for all populations at an increased risk of HIV-1. By 2019, about 626,000 PrEP users were in 77 countries globally, and 34% were in the African region. Although PrEP use globally increased by 69% in 2019 compared to 2018, some African countries did not even have a single PrEP user. It is projected that by the end of 2023, most PrEP users will be in the African and Americas regions, and 4.6 million will have received oral PrEP at least once that year [6]. Although oral PrEP is an effective strategy to reduce the risk of HIV-1 transmission, its effectiveness is highly dependent on users adhering to the drugs. Adherence to daily oral PrEP has been a significant challenge for some population groups. Studies in some African countries revealed that the adherence rate was higher in participants who did not seroconvert than in those who seroconverted [7]. The reasons given for non-adherence include low-risk perception, side effects, stigma, substance abuse, depression, and dosing regimen incompatibility. Low continuation rates for people initiated to PrEP have also been reported [8,9,10]. The availability of long-acting injectable PrEP that eliminates the need for a daily pill may increase PrEP uptake and adherence in people who struggle to adhere to oral PrEP [10,11].

In 2020, the HIV prevention clinical trials, HIV Prevention Trials Network (HPTN) 083 and 084 reported that long-acting cabotegravir (CAB-LA) for HIV-1 prevention was statistically superior to daily oral TDF/FTC for PrEP in cisgender men and transgender women (TGW) who have sex with men, and cisgender women. HPTN 083 revealed a 66% reduction in HIV-1 transmission in participants taking CAB-LA compared to those taking oral PrEP, while HPTN 084 reported an 89% risk reduction. Because clinical efficacy may not translate to real-world effectiveness, implementation will be vital to ensuring that the long-acting injectables reach those who desire to use them [8]. A study in China revealed that long-acting injectables would increase the proportion of MSM interested in PrEP by 24.5% over oral PrEP alone [9]. Another study conducted among the United States of America (USA) and African women revealed that greater than 75% of participants rated long-acting injectables as very acceptable. The study concluded that women’s demand for a long-acting injectable PrEP may be greater in African than in USA settings [10]. A systematic review and meta-analysis revealed that CAB-LA has satisfactory safety and pharmacokinetic profiles with similar safety profiles to oral PrEP [11]. The USA’s Food and Drug Administration (FDA) approved CAB-LA for use in at-risk adults and adolescents weighing at least 35 kg for PrEP to reduce the risk of sexually acquired HIV-1 in December 2021. The drug is given first as two initial injections administered one month apart, and then every two months after that. Patients can either start their treatment with the injection or oral cabotegravir for four weeks to assess the tolerability of the drug. The drug has also been approved in Canada, and the WHO is currently developing guidelines on offering CAB-LA for PrEP [12] In this review, we will discuss the implementation challenges to the successful roll-out of CAB-LA in Africa. We will further discuss measures to address these implementation challenges.

## 2. Implementation Challenges

To maximize the success of the roll-out of CAB-LA in Africa, there is a need to determine the possible implementation challenges. These implementation challenges can be divided into the health system and client challenges.

### 2.1. Health-System Level Challenges

African countries usually prepare guidelines after the WHO recommends the use of a new drug [13]. Therefore, until the WHO issues a statement urging the use of CAB-LA for PrEP, few African countries will consider CAB-LA for PrEP. Furthermore, without WHO recommendations, it will not be easy to source funding for CAB-LA from international organizations. Although CAB-LA for PrEP guidance is expected soon from the WHO, African countries will still have to consider the cost and cost-effectiveness of implementing CAB-LA for PrEP. CAB-LA currently costs $3700 per vial in the USA, an amount that can be used to buy oral PrEP for almost seven years for an individual in Africa [14]. Since the price of CAB-LA is likely to remain high for several years due to patent protections, African countries and medical insurance companies may be less willing to implement CAB-LA for PrEP of HIV due to budgetary constraints [14]. Since CAB-LA requires refrigeration during transportation, this may increase the cost of CAB-LA implementation.

Moreover, additional laboratory tests like liver function tests which are rarely done in patients on oral PrEP may further increase the cost of CAB-LA [15]. In addition, a steady supply chain of CAB-LA may be challenging to maintain for low-to-medium income countries (LMICs) during the patent protection period because manufacturers may prioritize high-income countries (HICs), as what initially happened to the COVID-19 vaccines [16]. Healthcare workers providing CAB-LA at facilities that do not have on-site pharmacists may find it challenging to manage medication supplies [17].

Furthermore, LMICs will need to invest in healthcare infrastructure before rolling out CAB-LA for PrEP of HIV-1. Since CAB-LA will need to be administered in the gluteal region [15], healthcare facilities may need to provide more private rooms for its administration, which will increase the initial investment for infrastructure to implement CAB-LA for PrEP of HIV-1. Clean needles and refrigerators at the healthcare facilities will be required to maintain the cold chain of CAB-LA [15]. This may pose a challenge since several healthcare facilities, especially those in rural areas, do not have refrigerators and reliable sources of power supply for the refrigerators. In addition, even urban healthcare facilities with refrigerators may be unable to maintain the cold chain due to frequent power cuts in African countries like South Africa and Tanzania [18,19]. Healthcare providers must be trained to identify clients eligible for CAB-LA, prescribe the drug, and administer it. Providing educational programs on CAB-LA evidence to healthcare providers will require more funding from LMICs with competing health priorities.

More healthcare providers may need to be employed since CAB-LA will be administered by injection. However, few healthcare providers can offer PrEP and administer injections in sub-Saharan Africa [20]. Clients requiring CAB-LA may need more consultation time to determine their eligibility. Patients taking antimycobacterial drugs like rifampin and rifabutin, and anticonvulsants like carbamazepine, phenobarbital, and phenytoin will be ineligible [15]. Convincing eligible clients to have CAB-LA while children, pregnant women, and breastfeeding mothers are excluded from using it may pose a challenge in communities that are already suspicious of vaccines and other medications from HICs [21]. In some African countries, patients are required to collect their chronic medication from one healthcare facility. Suppose this is implemented in the provision of CAB-LA, it may result in some clients discontinuing it whenever they change their residential places as a result of job transfers or family status changes. Several African countries lack a rapid response system for clients’ missed visits, which may affect their retention in CAB-LA care [7].

### 2.2. Client Challenges

Most of the challenges clients of CAB-LA may face are not specific to PrEP of HIV-1. Clients may fail to utilize CAB-LA because of a lack of knowledge about the drug. Suppose CAB-LA is offered at selected healthcare facilities, it may reduce its uptake since some clients who stay far away from the selected healthcare facilities may not have the money to travel to the facilities [22]. Even though clients may be interested in a long-acting PrEP regimen, the injection may be painful, leading to less interest in continuing with the method. Other side effects that may diminish interest in CAB-LA include hypersensitivity reactions, hepatotoxicity, depression, and weight gain [15]. Stigma remains a challenge for people infected and affected by HIV-1. Not taking medications daily may reduce stigma for some people. However, receiving an injection at a healthcare facility or department associated with HIV may potentially lead to clients being labeled as being HIV-positive, resulting in them discontinuing the injection. Although the acceptability of long-acting injectables is expected to be high, some clients may be less willing to use them since they will be administered by healthcare providers, leading to a perceived loss of control in their care, which they enjoy when taking oral PrEP. Clients taking CAB-LA may not receive the partner, family, or community support they require to continue with the medication due to a lack of knowledge. Clients are also more likely to discontinue CAB-LA if they are required to visit the healthcare facilities for frequent blood tests to monitor the hepatotoxicity of the injection. In addition, if the CAB-LA administration is not aligned to the provision of other health services, its uptake will likely be low. Furthermore, considering that most people in Africa are poor if CAB-LA is offered to clients at market value, most of them will not be able to afford it, resulting in a low uptake [10].

## 3. Measures to Address the Implementation Challenges

Although there are several implementation challenges to the successful roll-out of CAB-LA in Africa, several measures can be taken to address these challenges. These measures should be informed by lessons from the successful implementation of oral PrEP, ART, and immunization programs in the continent.

### 3.1. Recommendations and Demonstration Projects

Since most African countries develop guidelines after the WHO has published recommendations on the use of medications [13], it is vital that the WHO publishes its recommendations on the use of CAB-LA as a matter of urgency. This will ensure that international organizations and donor countries will start sponsoring the adoption of CAB-LA in African countries. This will also ensure that African countries will develop guidelines for implementing CAB-LA for PrEP of HIV-1. Demonstration projects should also be conducted in several African countries to inform the development of policies and standardized procedures for implementing CAB-LA for PrEP of HIV-1.

### 3.2. Cost and Availability of CAB-LA

For CAB-LA to be affordable to African countries, patent protection on the drug should be waived. This will allow for the production of generic products of CAB-LA, thereby ensuring a steady drug supply to African countries and other developing countries at lower costs [14]. The cost of CAB-LA to African countries may also be reduced by subsidization of the drug by international organizations such as Global Fund, PEPFAR, and Bill Gates Foundation; manufacturers; and HICs. Costs to African countries may also be reduced if they come together and buy the drug as a team as this will allow them to negotiate for more significant discounts from the manufacturers. The cost-effectiveness of CAB-LA can be increased by limiting access to clients who have difficulties maintaining adequate adherence. However, this will reduce availability to the general population.

### 3.3. Cold Chain Issues

Since CAB-LA requires refrigeration during transportation and storage, African countries should utilize immunization refrigerators to store the drug. This will ensure that the cold chain is maintained. For drug transportation, African countries should approach the private sector for refrigerated vehicles. At the same time, the military may come in to provide the logistics of transporting the drug to remote health facilities since they usually can reach such areas. Although power supply is unavailable or frequently interrupted in rural and urban areas in most African countries, renewable energy sources such as solar and wind energy may help address this problem [23].

### 3.4. Accessibility of CAB-LA

To ensure that CAB-LA is available to everyone who needs it, there should be an integration of CAB-LA administration into existing services such as sexually transmitted infections treatment, family planning, antenatal care, and postnatal care. Apart from increasing accessibility, it will also reduce initial infrastructure investment requirements and the stigma associated with the injection. CAB-LA provision should also be decentralized to primary care clinics since these are nearer to where the clients live, and this will reduce traveling expenses for clients. In addition, CAB-LA should also be offered for free in African countries to increase its utilization by people at high risk who need it but cannot afford it. CAB-LA should be provided at delivery locations outside the traditional healthcare facilities [24,25]. These delivery locations should include food banks and pharmacies [26]. Furthermore, after tolerability and safety have been ensured in some clients, they should be allowed to administer the drug themselves at home using microneedle arrays, provided they have the facilities to maintain the cold chain [18]. A bilayer microarray patch that can be used for the intradermal self-administration of CAB-LA is currently under development. If it is proven to be equivalent to the parenteral route, the patch may potentially increase the accessibility of CAB-LA since it will not require trained healthcare providers to administer it [27,28].

### 3.5. Healthcare Provider Training

The training of healthcare providers on the safe administration of parenteral medicines is important as it prevents cross-infection among the healthcare providers and patients. Training can also reduce errors during medicine administration, which may help prevent severe side effects on patients [29]. To reduce the cost of training healthcare providers in African countries, a few providers can be trained on the prescription, administration, and the side effects of CAB-LA. The trained healthcare providers will then train their colleagues in their regions at their facilities, without needing them to travel to big cities for the training [20].

### 3.6. Task Shifting

In several African countries, only doctors and nurses are allowed to administer injection drugs, while only doctors are allowed to prescribe injection drugs [20]. If the status quo is maintained, this will mean that most healthcare facilities will be unable to provide CAB-LA to clients. To address this, the prescribing and administration of CAB-LA should be delegated to lower-level cadres after adequate training. This will increase the availability and accessibility of CAB-LA to most clients on the continent.

### 3.7. Community Engagement and Client Education

Community engagement and client education are both important for medication adherence. They also promote mutual accountability and understanding between clients and healthcare providers. Informed and engaged clients are in a better position to make informed decisions about their healthcare options [30]. Proper steps should be put in place while preparing to implement CAB-LA for PrEP of HIV-1. All stakeholders, including community members, should be engaged to deliver messages around CAB-LA. Different promotional platforms such as television commercials, billboards, social media, and direct outreach programs should be used to promote the drugs and address misinformation about the drug. Educating communities about CAB-LA will make clients feel confident to report side effects, reduce myths about the drug, and reduce the stigma associated with taking the medication and being a member of key populations such as MSM, people who inject drugs, transgender people, and sex workers, thereby improving uptake and adherence [18].

### 3.8. Adherence Promotion

Adherence to CAB-LA may still be caused by the same factors that cause non-adherence to oral PrEP such as low-risk perception, side effects, and stigma. However, to improve adherence among clients on CAB-LA, clients should be well informed about the side effects they should expect. In addition, the burden on clients receiving CAB-LA should be reduced, which should involve reducing the frequency of laboratory tests to a bare minimum. Moreover, there should be a rapid response system for missed visits at all healthcare facilities offering CAB-LA, and ts should include voice calls or text messages via cell phones [13]. The microarray patch under development that can be used for the intradermal self-administration of CAB-LA may also improve adherence since it will be pain-free and will not be requiring healthcare workers for administration [28].

## 4. Conclusions

Although oral PrEP is an effective strategy to reduce the risk of HIV-1 transmission, its effectiveness is highly dependent on users adhering to the drugs. Low continuation rates for people initiated on PrEP have been reported. The availability of long-acting injectable PrEP that eliminates the need for a daily pill may increase PrEP uptake and adherence in people who struggle to adhere to oral PrEP. The development of the microarray patch may further improve accessibility, uptake, and adherence to CAB-LA. The USA’s FDA approved CAB-LA for PrEP of HIV-1 in December 2021. Although most people in Africa may accept using CAB-LA for PrEP of HIV-1, its successful implementation may be hindered by the health system and client challenges. However, these challenges can be addressed by several measures emanating from lessons learned from the successful implementation of ART, oral PrEP, and immunization in the continent. Since CAB-LA implementation is still in its infancy, a meta-analysis to support our ideas was not possible because there were no studies conducted on the topic yet.

## Data Availability

Not applicable.
